# Traditional tonifying polyherbal infusion, *Jatu-Phala-Tiga*, exerts antioxidant activities and extends lifespan of *Caenorhabditis elegans*

**DOI:** 10.1186/s12906-019-2626-1

**Published:** 2019-08-13

**Authors:** Palika Wetchakul, Jo Aan Goon, Ademola Ezekiel Adekoya, Opeyemi Joshua Olatunji, Sutticha Ruangchuay, Patcharawalai Jaisamut, Acharaporn Issuriya, Nongluk Kunworarath, Surasak Limsuwan, Sasitorn Chusri

**Affiliations:** 1 Faculty of Traditional Thai Medicine, Prince of Songkla University, Hat Yai, Songkhla 90110 Thailand; 2 Department of Biochemistry, Faculty of Medicine, Universiti Kebangsaan Malaysia, Kuala Lumpur, 56000 Malaysia; 3 Department of Physiology, Faculty of Science, Prince of Songkla University, Hat Yai, Songkhla 90110 Thailand; 4 Natural Product Research Center of Excellence, Prince of Songkla University, Hat Yai, Songkhla 90110 Thailand

**Keywords:** Antioxidants, Polyherbal formula, Tonifying agents, Herbal tonic, *Caenorhabditis elegans*

## Abstract

**Background:**

The imbalance between the generation of free radicals and natural cellular antioxidant defenses, known as oxidative stress, can cause oxidation of biomolecules and further contribute to aging-associated diseases. The purpose of this study was to evaluate the antioxidant capacities of Thai traditional tonifying preparation, Jatu-Phala-Tiga (JPT) and its herbal ingredients consisting of *Phyllanthus emblica*, *Terminalia arjuna*, *Terminalia chebula*, and *Terminalia bellirica* and further assess its effect on longevity.

**Method:**

Antioxidant activities of various extracts obtained from JPT and its herbal components were carried out using well-established methods including metal chelating, free radical scavenging, and ferric reducing antioxidant power assays. Qualitative analysis of the chemical composition from JPT water extract was done by high-performance liquid chromatography tandem with electrospray ionisation mass spectrometry. The effect of JPT water extract on the lifespan of *Caenorhabditis elegans* were additionally described.

**Results:**

Among the extracts, JPT water extract exerted remarkable antioxidant activities as compared to the extracts from other solvents and individual constituting plant extract. JPT water extract was found to possess the highest metal chelating activity, with an IC_50_ value of 1.75 ± 0.05 mg/mL. Moreover, it exhibited remarkable scavenging activities towards DPPH, ABTS, and superoxide anion radicals, with IC_50_ values of 0.31 ± 0.02, 0.308 ± 0.004, and 0.055 ± 0.002 mg/mL, respectively. The ORAC and FRAP values of JPT water extract were 40.338 ± 2.273 μM of Trolox/μg of extract and 23.07 ± 1.84 mM FeSO_4_/mg sample, respectively. Several well-known antioxidant-related compounds including amaronols, quinic acid, gallic acid, fertaric acid, kurigalin, amlaic acid, isoterchebin, chebulagic acid, ginkgolide C, chebulinic acid, ellagic acid, and rutin were found in this extract. Treatment with JPT water extract at 1 and 5 mg/mL increased *C. elegans* lifespan under normal growth condition (7.26 ± 0.65 vs. 10.4 0± 0.75 (*p* < 0.01) and 10.00 ± 0.73 (*p* < 0.01) days, respectively).

**Conclusions:**

The results indicated that JPT and its herbal ingredients exhibited strong antioxidant activities, in particular the water extract of the polyherbal tonic. These findings rationalize further investigation in JPT infusion as a promising agent for anti-aging and oxidative stress prevention.

## Background

Aerobic respiration utilizes oxygen in its combustion process. Part of the oxygen utilized for respiration generates reactive oxygen species (ROSs) that includes: singleton oxygen, superoxide anion, and hydroxyl, carbonate, and alkoxyl radicals. The oxidative burst of phagocytes and the enzymatic system like the xanthine oxidase and the cytochrome P-450 can also be a source of endogenous generation of ROS [1]. Several studies have clearly demonstrated that aging and the development of various degenerative diseases in humans such as diabetes, arthritis, cancer, and cardiovascular complications could be directly linked to oxidative imbalance [2].

Previous studies have shown that secondary compounds from medicinal plants [3], fruits [4], and vegetables [5] which exhibit antioxidant potential are effective in increasing lifespan of model animals and reversing age-related deficits. For example, polyphenols and proanthocyanidins isolated from blueberries have been shown to confer remarkable antioxidant activities, protect cells against oxidative stress, and extended *C. elegans* lifespan [6]. Some polyherbal formulas such as PHE, an Ayurvedic polyherbal extract which comprises of six herbs [7] and SC100, a Chinese traditional polyherbal extract which consists of *Astragalus membranaceus* root, *Pterocarpus marsupium* bark, pine bark, oligo-proanthocyanidins, and L-theanine [3] were found to elicit antioxidant and anti-aging effects. PHE enhanced stress tolerance and increased the mean lifespan of *C. elegans*, while SC100 had notable effect on the mean life longevity of *Drosophila melanogaster*. These scientific evidences clearly indicated that plant-derived compounds, particularly phenolic compounds, may serve as an external source of antioxidants, which can assist in dealing with oxidative stress and aging-related diseases.

The ethanol extract of a Thai traditional polyherbal tonic, Jatu-Phala-Tiga (In *Thai*: *Jatu* means four; *Phala* means fruits; *Tiga* means benefits or usefulness) has been found to possess remarkable antioxidant activity [8], its water infusion which is widely used in Thai traditional medicine has never been examined. This study was therefore aimed at ascertaining the anti-oxidative capacity of the water infusion of the Thai polyherbal tonic, Jatu-Phala-Tiga (JPT) and its constituting components (*Terminalia arjuna*, *Terminalia chebula*, *Terminalia bellirica*, and *Phyllanthus emblica*) as well as the effect of the infusion on the lifespan of *Caenorhabditis elegans*.

## Methods

### Physico-chemical determination of four botanical ingredients of JPT

The fruits of *Phyllanthus emblica* L. (Phyllanthaceae), *Terminalia chebula* Retz. (Combretaceae), *Terminalia bellirica* (Gaertn.) Roxb. (Combretaceae), and *Terminalia arjuna* (Roxb. Ex DC.) Wight & Arn. (Combretaceae) were purchased in May 2014 from a licensed traditional medical drug store, Triburi Orsot, in Songkla, Thailand. The formal identification of the plant material used in this study were undertaken by a botanist, Assistant Professor Dr. Katesarin Maneenoon. Voucher specimens of the plants (*P. emblica*; MTM08–72, *T. chebula*; MTM08–92, *T. arjuna*; MTM08–90, *T. bellirica*; MTM08–91) were deposited at the herbarium of Materia Medica, Faculty of Traditional Thai Medicine, Prince of Songkla University.

The plant materials were cleaned with running tap water, dried in an air blowing thermostatic oven at 60 °C for 72 h, grounded into powder and then kept in an airtight container at 4 °C until further use. Physico-chemical parameters of the plants such as total ash, acid insoluble ash, loss on drying, and extractive values (ethanol-soluble extractive, water-soluble extractive, and 70% ethanol-soluble extractive) were determined in triplicate as per the procedure described in Thai Herbal Pharmacopoeia [9] and the Ayurvedic Pharmacopoeia of India [10].

### Preparation of JPT and four botanical extracts

The dried powders of plant ingredients were sieved through a 2 mm screen, accurately weighed, and then mixed together in a 1:1:1:1 ratio to obtain JPT based on the procedure described in the Thai Pharmaceutical Textbook. Two-hundred grams of JPT and its individual plant ingredient powder were individually macerated with 600 mL of ethyl acetate, or ethanol at ambient temperature for 7 days. Water extracts were prepared according to the generally practiced traditional preparation method for JPT. Each herbal powder (1.25 g) was soaked in 100 mL of hot distilled water (97 ± 2 °C) for 3 min. Each infusion was then filtered through Whatman No. 1 filter paper and either dried using a vacuum rotary evaporator (ethyl acetate and ethanol extracts) or freeze-dried (water extracts). All the plant extracts were stored at − 20 °C until further experiments. The extraction yield of each plant extract was calculated as weight percent (% w/w) using the equation below:

$$ \mathrm{Extraction}\ \mathrm{yield}\ \left(\%\right)=\left(\mathrm{weight}\ \mathrm{of}\ \mathrm{the}\ \mathrm{dry}\ \mathrm{extract}\times \kern0.37em 100\right)/\mathrm{weight}\ \mathrm{of}\ \mathrm{the}\ \mathrm{initial}\ \mathrm{dry}\ \mathrm{material} $$

### Antioxidant-related chemical constituents

Each plant extract from different solvents were assessed for the TPC quantification using Folin-Ciocalteu method [11, 12] with slight modifications. In brief, 120 μL of each plant extract (2.5 mg/mL) was mixed with 1 mL of 10-fold diluted Folin-Ciocalteu reagent for 5 min, followed by adding 1 mL of 20%w/v sodium carbonate solution (Ajax Finechem, New Zealand). The solution was thoroughly mixed and allowed to stand for 90 min in the dark at room temperature. The absorbance of the resultant solution was measured at 725 nm (Sunrise™ Microplate reader, Tecan Group Ltd., Switzerland). The TPC value was expressed in terms of milligrams of gallic acid (Sigma-Aldrich Chemie, Germany) equivalents per gram of extract through the calibration curve of gallic acid.

The TFC in the plant extracts was conducted based on the aluminium chloride colorimetric method as proposed previously [11]. Briefly, 50 μL of the plant extract (2.5 mg/mL) was combined with 300 μL of 5% (w/v) sodium nitrite (Ajax Finechem, New Zealand), 300 μL of 10% (w/v) aluminium trichloride (Ajax Finechem, New Zealand), and 4 mL of distilled water. Subsequently, the solution was thoroughly mixed and incubated for 6 min at room temperature. The reaction was stopped with 2 mL of 1 M sodium hydroxide and then sterile distilled water was added to bring to a final volume of 10 mL and allowed to stand for 10 min. The absorbance of the solution was spectrophotometrically read at 510 nm. The TFC value was calculated using the calibration curve of catechin (Sigma-Aldrich Chemie, Germany) and expressed as milligrams of catechin equivalents per gram of extract.

Preliminary profiling of the chemical constituents of JPT was conducted with an Agilent 1290 Infinity ultra-high-performance liquid chromatography system-tandem mass spectrometry method with electrospray ionization [13]. The optimization of the instrument settings was as follows: gas temperature was 325 °C at a flow rate of 13 L/min, nitrogen was used as the nebulizer at 35 psi and the capillary voltage was 3.5 kV. The mobile phase consisted of a linear gradient of 0.1% (v/v) acetic acid in ultrapure water (A) and acetonitrile (B): 0 to 5.0 min, 5% B (v/v); 5.0 to 38.0 min, 42% B (v/v); 38.0 to 45 min, 5% B (v/v). The flow rate was 0.2 mL/min and the injected volume was 5 μL. Putative compounds were processed using Agilent Mass Hunter Workstation software (Version B.04.00), Agilent MSC software (Version B.07.00) and the online METLIN database. The accuracy error threshold was set at ≤5 ppm.

### Metal chelating capacity

The ability of the polyherbal extracts to chelate ferrous ions was determined based on the colorimetric method [14]. Two-hundred fifty microliters of two-fold dilution of each plant extract at a concentration range of 0.03–62.50 mg/mL were mixed with distilled water (800 μL) and 2 mM of iron (II) chloride (25 μL). The reaction was initiated by adding 5 mM of ferrozine (25 μL) and incubated at room temperature for 10 min. The increase in absorbance of stable ferrous-ferrozine complex was detected at 562 nm. Ethylenediaminetetraacetic acid (EDTA) was used as a positive control. The percentage chelating capacity of the complex formation was calculated as:

$$ \mathrm{MCA}\ \left(\%\right)=\left(\left({\mathrm{Ab}}_{\mathrm{control}}-{\mathrm{Ab}\mathrm{s}}_{\mathrm{sample}}\right)\times \kern0.37em 100\right)/{\mathrm{Ab}\mathrm{s}}_{\mathrm{control}} $$

The metal chelating activity (MCA) of the plant extracts was presented as the concentrations providing 50% inhibition of ferrous ion ferrozine complex (IC_50_; mg/mL).

### Free radical scavenging activities

#### Mixed-mode assays: DPPH and ABTS radical scavenging assays

The radical scavenging effects of the plant extract were assessed using DPPH and ABTS radical scavenging activities [11].

For the radical scavenging ability of the extracts towards DPPH radical, samples were initially diluted two-fold and aliquots (20 μL) of each sample at concentrations between 1.22 to 2500 μg/mL were placed in a 96-well plate containing 80 μM DPPH in ethanol solution (180 μL). The plate was properly shaken and incubated in the dark at ambient temperature for 30 min. The absorbance of the solution was read at 520 nm.

To generate ABTS^+^, 2 mM of ABTS and 2.45 mM of potassium persulfate were mixed together at a volume ratio of 1:1. The mixture was allowed to stand in the dark at room temperature for 16 h. The absorbance of the solution was maintained at 0.70 ± 0.05 at 734 nm with ethanol. A two-fold serial dilution (10 μL) of each extract at concentrations of 1.22 to 2500 μg/mL was added to 1 mL of ABTS^+^ solution and allowed to stand for 6 min. The absorbance was read at 734 nm.

Trolox was used as a reference compound. The scavenging activity of the extracts was expressed as the concentration that caused 50% inhibition of DPPH/ABTS+ radicals (IC_50_; mg/mL). The percentage of DPPH/ABTS^+^ based-scavenging activities was calculated using equation below:

$$ \mathrm{Scavenging}\ \mathrm{activity}\ \left(\%\right)=\left(\left({\mathrm{Ab}}_{\mathrm{control}}-{\mathrm{Ab}\mathrm{s}}_{\mathrm{sample}}\right)\times \kern0.37em 100\right)/{\mathrm{Ab}\mathrm{s}}_{\mathrm{control}} $$

#### Single electron transfer-based assays: ferric-reducing antioxidant power (FRAP) assay and superoxide anion radical scavenging assay

FRAP activity of the plant extract was determined according to Peng and his colleagues [15]. FRAP working solution was freshly prepared by mixing 10 mL of 300 mM acetate buffer, 1 mL of 10 mM TPTZ solution, and 10 mL of 20 mM ferric chloride. One-hundred fifty microliters of each extract was diluted in ethanol to a concentration of 0.625 mg/mL and 1.35 mL of the FRAP solution was added and incubated at 37 °C in the dark for 30 min. An intense blue colour complex was formed by the reduction of ferric-tripyridyl triazine (TPTZ) complex to ferrous-TPTZ in the presence of electron donating antioxidants at low pH. The absorbance of the coloured product in the reaction mixture was measured at a wavelength of 596 nm. The reducing power of the extracts was estimated by an increase in absorbance of the reaction mixture. The reducing capacity was expressed as μM Fe_2_SO_4_/mg extract.

The superoxide anion radical scavenging activity was estimated by the reduction of nitroblue tetrazolium (NBT) method [16] with some modifications. The riboflavin/methionine/ illuminate system was used to generate superoxide anion radicals, which reduced NBT to form purple formazan (NBT^2+^). The reaction mixture contained 100 μL of NBT (400 μg/mL) and 0.4 mL of the solution consisting of a mixture of riboflavin (30 μg/mL), methionine (30 μg/mL), EDTA (20 μg/mL), and the plant extract at different concentrations (2-fold dilution; 4.88 to 156.25 μg/mL) diluted in 0.05 M phosphate buffer, pH 7.4. Photo-induced superoxide radicals were initiated with illumination of fluorescent lamps (20 W) at 25 °C for 25 min.

After incubation, the absorbance was measured at 560 nm. The ability of the plant extract to scavenge superoxide radical was defined as the concentrations that produced 50% inhibition of superoxide anion radicals (IC_50_; mg/mL). The percentage inhibition was calculated by plotting a graph of the absorbance against the corresponding concentrations. Catechin was used as a reference compound.

#### Hydrogen atom transfer-based assay; Peroxyl radical scavenging assay (oxygen radical antioxidant capacity (ORAC) assay)

The assay of with some modifications was used to evaluate antioxidant activity of the extracts against peroxyl radicals generated from thermal homolysis of 2,2′-Azobis (2-amidinopropane) dihydrochloride (AAPH). The working solutions consisting of 0.4 nM fluorescein, 153 mM AAPH, different concentrations of the extracts at concentrations of 0.2 to 100 μg/mL (2-fold dilution) and a reference compound (Trolox) were prepared in 75 mM phosphate buffer (pH 7.4). The reaction was carried out in a black round bottom 96-well microplate by mixing 25 μL of the plant extract or the reference compound with 150 μL of the fluorescein solution. After 30 min of incubation at 37 °C, 25 μL of AAPH solution was added to the solution. Fluorescence measurements of the solution was performed at an emission wavelength of 528 nm and an excitation wavelength of 485 nm every 5 min for 90 min. The antioxidant capacity was established based on Trolox equivalents per μg of the extract (μM of TE/μg of E) [17].

### *Caenorhabditis elegans* lifespan assay

#### *C. elegans* strain and maintenance

The wild type *C. elegans* strain (N2) was maintained and handled according to procedures described previously [18]. The nematodes were maintained at 20 °C on solid nematode growth medium (NGM) supplemented with heat-killed *Escherichia coli* OP50 as a food source following standard procedures and transferred into plates containing 40 μM 5-fluoro-29-deoxyuridine (FUdR; Sigma-Aldrich, St. Louis, MO, USA) to prevent the production of progeny. Age-synchronized animals (L4 stage) were used in all lifespan and stress assays. All assays were performed in three replicates with at least fifty animals per experiment.

#### Lifespan and oxidative stress assays

Since JPT infusion possessed notable in vitro antioxidant capacity, therefore we further evaluated whether this water extract influence the lifespan of *C. elegans* under normal conditions. The water extract was mixed with NGM containing FUDR to achieve final concentrations of 1, 5, 10, and 20 mg/mL. The worms were reared on the NGM agar plates at 20 °C and counted daily. Nematodes were recognized as dead and eliminated when the body is straight with no response to probing. The survival of nematodes was determined as the percentage of worms alive per total number of worms visualized under the microscope.

In addition to the lifespan extension assay described above, the effect of JPT in term of the factor which could improve the repairment of oxidative stress-damaged biomolecules were determined. The measurement of oxidative stress resistance in *C. elegans* by H_2_O_2_ as a ROS-inducing reagent was performed in NGM supplemented with the JPT water extract at 5 mg/mL. The concentration of H_2_O_2_ used to induce oxidative stress in *C. elegans* was calculated to be 1.4 mM because this concentration gave greater than 50% nematode survival compared with the control. The age-synchronized L4 nematodes were exposed to 1.4 mM H_2_O_2_ for 2 h and transferred to NGM plates containing the JPT water extract at 5 mg/mL, incubated at 20 °C, and the number of live and dead worms was monitored daily for all experiments [19].

### Statistical analyses

The results obtained in this study are expressed as mean ± standard deviation (SD) from triplicate assays. Differences among samples were assessed by one-way ANOVA followed by Duncan’s multiple test. All statistics were performed using the software package SPSS for Windows version 17.0 (SPSS, Chicago, USA). Mean and median lifespans of *C. elegans* were constructed using Kaplan-Meier survival curves and analyzed using the log-rank (Mantel-Cox) test.

## Results

### Pharmacognostic specification of JPT’s herbal components

The assessment of the quality of medicinal plants used as traditional remedies is of high importance. The pharmacognostic parameters of the plants *T. arjuna*, *T. chebula*, *T. bellirica* and *P. emblica* which are the constituents of the herbal remedy JPT were tested. *T. bellirica*, *T. chebula* and *P. emblica* were tested in accordance with the Thai herbal pharmacopoeia; while the parameters explored for *T. arjuna* were according to the Ayurvedic herbal pharmacopoeia (Table 1). The total ash, acid-insoluble ash, and the percentage weight lost on drying indicates the existence of impurities in medicinal plant materials such as the presence of carbonates, phosphates, silicates, or moisture. Solvent-soluble extractive values are examined to estimate the amount of active constituents of the plant samples when extracted with a particular solvent. With exception of the total ash content obtained from *T. chebula*, the amount of foreign matters, total ash content, acid insoluble ash, and percentage of loss on drying of the tested plants were found to be within the limit as per the specification in the pharmacopoeias. With exception of *T. chebula*, the extractive values of the tested plants were found to meet the criteria required in the pharmacopoeias.

**Table 1:** Pharmacognostic specification of *Terminalia arjuna*, *Terminalia chebula*, *Terminalia bellirica*, and *Phyllanthus emblica*

Parameters	Content (% by weight)			
	*T. arjuna* ^a^	*T. chebula* ^b^	*T. bellirica* ^b^	*P. emblica* ^b^
Foreign matter	ND	0.02	1.91	0.95
Total ash	3.43 ± 0.16	3.70 ± 0.23	4.69 ± 0.51	3.00 ± 0.1
Acid-insoluble ash	0.03 ± 0.02	0.07 ± 0.00	0.10 ± 0.05	0.03 ± 0.02
Loss on drying	NA	7.25 ± 0.07	7.24 ± 0.02	7.60 ± 0.04
Ethanol-soluble extractive	38.67 ± 0.56	16.48 ± 1.29	22.27 ± 1.52	19.38 ± 0.39
70% ethanol-soluble extractive	NA	22.29 ± 0.37	35.75 ± 1.39	NA
Water-soluble extractive	42.15 ± 0.63	20.57 ± 1.97	28.93 ± 1.11	32.19 ± 1.11
Determination of tannins	NA	18.36 ± 0.53	21.61 ± 0.02	21.61 ± 0.02

*ND* Not detected, *NA* Not applicable,

^a^The parameters were tested according to Ayurvedic herbal pharmacopoeia

^b^The parameters were tested according to Thai herbal pharmacopoeia

### In vitro metal chelating and free radical scavenging activities of JPT and its herbal ingredients

The results (Table 2) indicated that among the three solvents used for extraction, ethanol gave the highest extraction yield for JPT, *T. arjuna, T. chebula* and *P. emblica* (in g of dried extract /100 g of dried plant materials), while ethyl acetate gave the lowest yield for all medicinal plants.

**Table 2:** Effects of extracting solvents on extraction yields of Jatu-Phala-Tiga and its herbal components

Plant materials	Extraction yield (g/100 g of dried plant materials)		
	Ethyl acetate	Ethanol	Water
Jatu-Phala-Tiga	1.32	10.85	9.83
*Terminalia arjuna*	1.56	13.30	8.08
*Terminalia chebula*	1.78	18.18	10.42
*Terminalia bellirica*	0.81	3.46	15.17
*Phyllanthus emblica*	0.99	18.63	10.75

Results from Table 3 shows that the water extract of JPT had the highest metal chelating ability with an IC_50_ value of 1.75 ± 0.05 mg/mL, whereas its ethanol extract possessed a remarkable reducing power with the FRAP value of 23.07 ± 1.84 mMFeSO_4_/mg. The water extracts also displayed the highest DPPH radical scavenging activity, while the ethanol extract displayed significant inhibitory activity towards ABTS radicals with IC_50_ values of 0.31 ± 0.02 mg/mL and 0.27 ± 0.01 mg/mL, respectively. The ethyl acetate extract of JPT showed much lower antioxidant capacities than the ethanol or water extracts. However, the ethyl acetate extract had the highest TFC value, followed by the ethanol extract, and then the water extract with values of 78.25 ± 7.91, 68.06 ± 1.65, and 64.92 ± 3.65 mg of catechin equivalent/ g of the extracts, respectively. Table 3 displays the total phenolic contents in milligram (mg) equivalent of gallic acid per gram (g) of the extracts. The values range from 253.01 ± 5.45 to 457.81 ± 13.24 mg gallic acid/g extract.

**Table 3:** Metal chelating activity (MCA), ferric reducing-antioxidant power (FRAP), free radical scavenging capacities, total phenolic content, and total flavonoid content of different extracts of Jatu-Phala-Tiga

Extracting solvents	MCA assay* (IC_50_; mg/mL)	FRAP assay (mM FeSO_4_/mg)	Radical scavenging properties** (IC_50_; mg/mL)		Total contents of (mg equivalence/g of extract)	
			DPPH	ABTS	Phenolics	Flavonoids
Ethyl acetate	48.62 ± 8.57^b^	14.22 ± 0.16^b^	0.99 ± 0.04^c^	0.59 ± 0.01^c^	350.20 ± 10.23^b^	78.25 ± 7.91^a^
Ethanol	216.09 ± 8.78^c^	23.07 ± 1.84^a^	0.41 ± 0.04^b^	0.27 ± 0.01^a^	457.81 ± 13.24^a^	68.06 ± 1.65^ab^
Water	1.75 ± 0.05^a^	16.81 ± 0.46^b^	0.31 ± 0.02^a^	0.31 ± 0.00^b^	253.01 ± 5.45^c^	64.92 ± 3.65^b^

*IC_50_ of EDTA (a positive control) was 0.021 ± 0.00 mg/mL

**IC_50_ of trolox obtained from DPPH and ABTS assays were 0.143 and 0.533 mg/mL, respectively

^a-c^Values in the same column with different superscripts are significantly different (*p* < 0.05)

As described in Table 4, the metal chelating ability of water extracts from all the plants were found to be the highest as compared to the extracts from the other extracting solvents. The water extracts of *T. arjuna* and *T. bellirica* additionally provided higher FRAP values compared to ethanol and ethyl acetate extracts. However, the ethanol extracts of JPTs’ herbal ingredients tend to present higher levels of ABTS and DPPH radical scavenging activities as well as total phenolic and flavonoid contents.

**Table 4:** Metal chelating activity (MCA), ferric reducing-antioxidant power (FRAP), free radical scavenging capacities, total phenolic content, and total flavonoid content of different extracts of *Phyllanthus emblica*, *Terminalia arjuna*, *Terminalia bellirica*, and *Terminalia chebula*

Medicinal plants	MCA assay (IC_50_; mg/mL)	FRAP assay (mM FeSO_4_/mg)	Radical scavenging properties (IC_50_; mg/mL)		Total contents of (mg equivalence/g of extract)	
			DPPH	ABTS	Phenolics	Flavonoids
*Phyllanthus emblica*	^ET^16.41±1.60^b^	21.50±1.51^a^	0.56±0.02^c^	0.27±0.02^a^	345.89±3.28^b^	41.55±1.63^c^
	^E^26.13±1.41^c^	14.55±0.72^b^	0.35±0.03^b^	0.53±0.00^c^	374.09±12.45^a^	87.35±1.65^a^
	^W^11.15±0.42^a^	16.81±0.46^b^	0.13±0.02^a^	0.32±0.01^b^	292.89±12.36^c^	77.47±6.24^b^
*Terminalia arjuna*	^ET^20.34±1.53^c^	15.26±0.19^c^	0.92±0.01^b^	0.21±0.01^a^	253.37±28.93^c^	95.04±4.21^a^
	^E^13.22±1.84^b^	16.43±0.73^b^	0.34±0.01^a^	1.94±0.24^a^	376.61±8.90^b^	42.80±1.90^c^
	^W^5.27±0.17^a^	21.12±0.19^a^	0.41±0.07^a^	0.25±0.02^b^	446.49±13.37^a^	70.41±2.59^b^
*Terminalia bellirica*	^ET^14.11±0.23^a^	18.50±0.40^c^	0.44±0.07^b^	0.23±0.01^b^	321.46±3.16^b^	60.21±1.08^b^
	^E^61.18±7.17^b^	21.72±0.87^b^	0.37±0.03^a^	0.19±0.02^a^	405.71±9.35^a^	58.02±2.62^b^
	^W^13.20±1.07^a^	23.37±0.85^a^	0.79±0.05^c^	0.26±0.00^b^	267.20±4.69^c^	84.62±5.98^a^
*Terminalia chebula*	^ET^60.97±4.09^c^	11.30±0.17^c^	0.50±0.06^b^	0.36±0.01^b^	380.20±7.70^ab^	44.37±3.29^c^
	^E^12.47±1.23^b^	19.16±0.31^a^	0.39±0.01^a^	0.21±0.01^a^	413.08±21.76^a^	85.00±5.43^a^
	^w^4.64±0.17^a^	12.25±0.18^b^	0.59±0.02^c^	0.55±0.01^c^	381.28±10.31^b^	54.10±1.18^b^

^a-c^Values in the same column with different superscripts are significantly different in the same medicinal plant (*p* < 0.05)**.** ^ET^ethyl acetate, ^E^ethanol, or ^W^water.

Furthermore, *T. chebula* water extract displayed the highest metal chelating activity with an IC_50_ of 4.64 ± 0.17 mg/mL, while *T. bellirica* showed the strongest capability to reduce Fe^3+^ to Fe^2+^ (23.37 ± 0.85 mM FeSO_4_/mg). *P. emblica* water extract and *T. bellirica* ethanol extract had remarkable DPPH and ABTS free radical scavenging activities with the IC_50_ values of 0.13 ± 0.02 and 0.19 ± 0.02 mg/mL, respectively. The water extract of *T. arjuna* showed the highest total phenolic content (446.49 ± 13.37 mg of gallic acid equivalent/ g of the extracts) and its ethyl acetate extract possessed the highest total flavonoid content (95.04 ± 4.21 mg of gallic acid equivalent/ g of the extracts).

### Qualitative analysis of antioxidant-related constituents

Qualitative analysis on compounds in the JPT infusion using HPLC-DAD-ESI-MS in negative mode revealed the known antioxidant-related constituents as amaronols (RT = 1.78) quinic acid (RT = 1.91), gallic acid (RT = 4.32), fertaric acid (RT = 7.63), kurigalin (RT = 15.64), amlaic acid (RT = 17.17), quercetin 3′-O-glucuronide (RT = 19.48), isoterchebin (RT = 23.69), chebulagic acid (RT = 24.35), ginkgolide C (RT = 26.88), furocoumarinic acid glucoside (RT = 27.05), chebulinic acid (RT = 29.82), ellagic acid (RT = 31.42), and rutin (RT = 31.59). In addition, several polyphenols have also been found in this water extract such as 4-glucogallic acid, 1,2′-Di-O-galloylhamamelofuranose, 3-O-galloylhamamelitannin, 2-O-caffeoylglucarate, and furocoumarinic acid glucoside.

### Superoxide anion and peroxyl radical scavenging activities

**Fig. 1:**
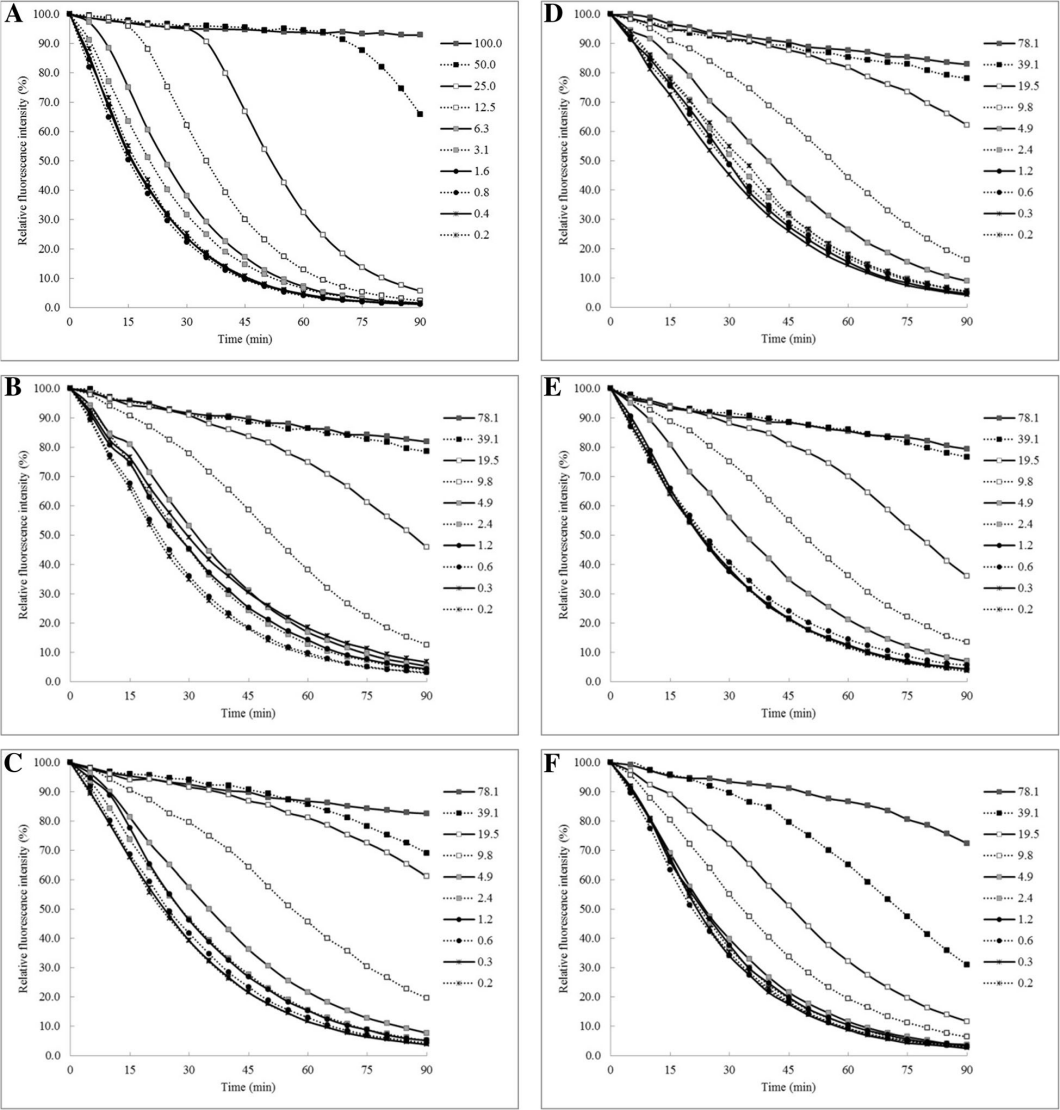
Fluorescence decay curves induced by AAPH in the presence of trolox (a positive control: **a**) at 100–0.2 μg/mL and water extracts of Jatu-Phala-Tiga (**b**), *Phyllanthus emblica* (**c**) *Terminalia arjuna* (**d**), *Terminalia bellirica* (**e**), and *Terminalia chebula* (**f**) at 78–0.2 μg/mL

**Fig. 2:**
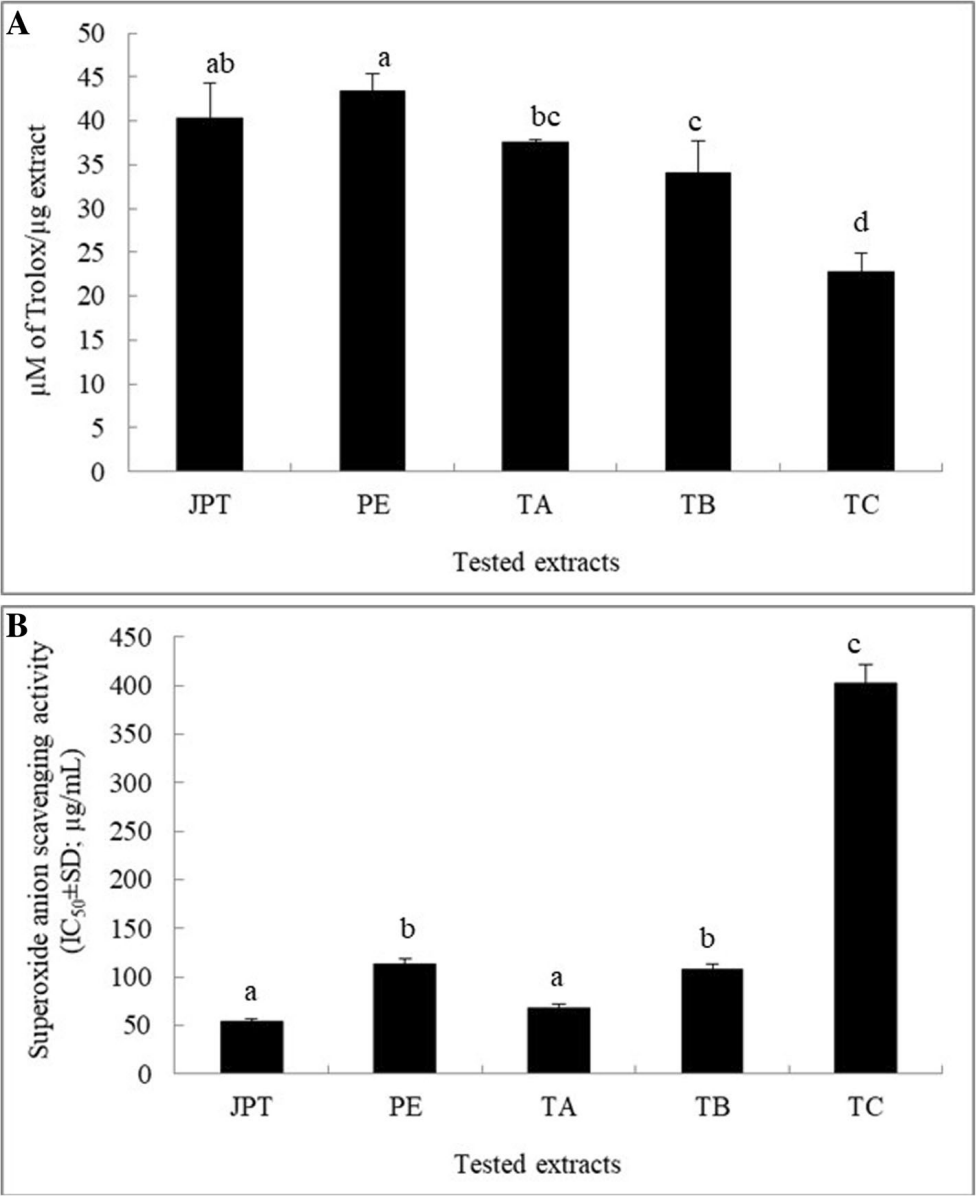
Effects of *Jatu-Phala-Tiga* (JPT), *Phyllanthus emblica* (PE), *Terminalia arjuna* (TA), *Terminalia bellirica* (TB), and *Terminalia chebula* (TC) water extracts on production of peroxyl radicals (**a**) and superoxide anion radicals (**b**). All values are presented as the means± SD. Bars with different letters indicate statistically significant differences among groups at *p* < 0.05 by one-way ANOVA

As shown in Fig. 1, the tested extracts scavenged peroxyl radicals in a concentration-dependent manner as indicated by the inhibition of fluorescence decay. In this study, the water extracts of *P. emblica*, and JPT exhibited remarkable peroxyl radical scavenging properties with ORAC values of 43.33 ± 2.02 and 40.34 ± 3.94 μM Trolox/μg of extract, respectively (Fig. 2a). The IC_50_ values of JPT and *T. arjuna* extracts toward superoxide anion radical were 54.7 ± 2.2 and 67.5 ± 4.6 μg/mL, respectively, which clearly indicated their greater efficiency as superoxide anion scavengers (Fig. 2b).

### Effect of JPT water extract on lifespan extension

**Fig. 3:**
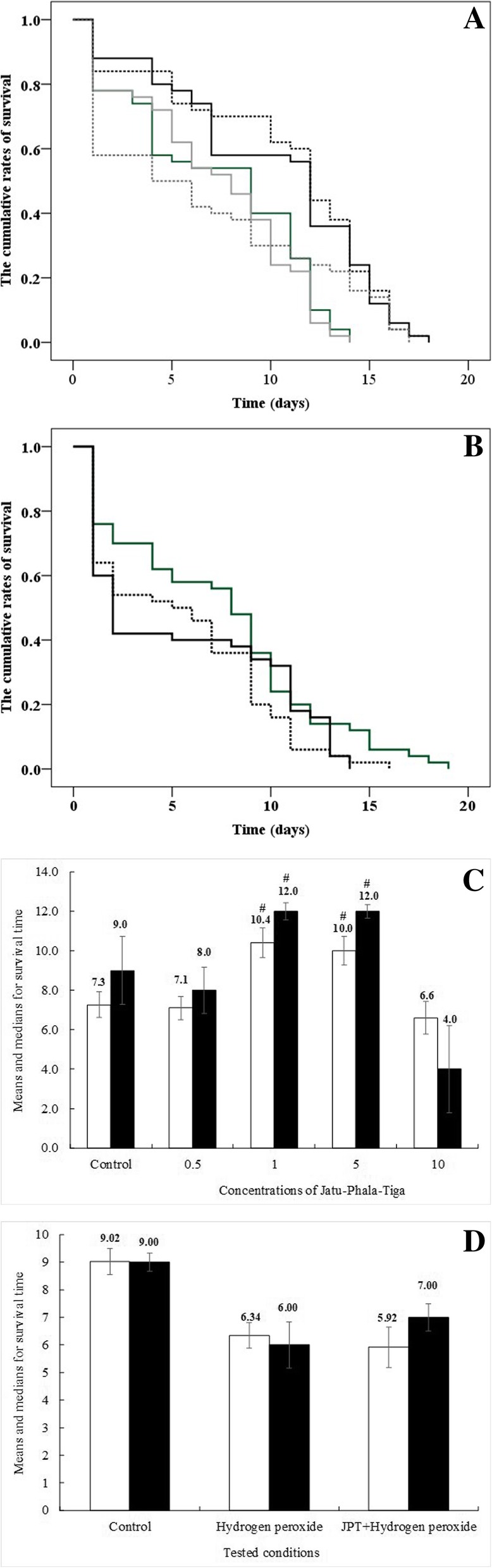
Kaplan-Meier survival estimates the effect of different concentrations of Jatu-Phala-Tiga (JPT) water extract on the means and median lifespan of wild-type *Caenorhabditis elegans* under (**a**) the normal condition (untreated control; green line, treated with JPT at 0.5 mg/mL; grey line, 1 mg/mL; broken black line, 5 mg/mL; black line, and 10 mg/mL; dark broken grey line,) and (**b**) H_2_O_2_-induced oxidative stress condition (untreated control; green line, H_2_O_2_ exposed control; broken black line, H_2_O_2_ exposed and treated with 5 mg/mL of JPT; black line). The mean lifespan (white bars) and the median survival time (black bars) of the nematodes (*n* = 50–100) under the normal condition (**c**) and H_2_O_2_-induced oxidative stress condition (**d**) were calculated from three independent experiments. *p* < 0.05 were considered as significantly different from untreated control group by the Log-rank (Mantel-Cox) test

The effect of the different concentrations of JPT water extract was determined on the mean lifespan of wild type *C. elegans*. The results as shown in Fig. 3 revealed that the worm has a mean lifespan of 7.26 ± 0.65 days at normal conditions. The mean lifespan of *C. elegans* was significantly increased to 10.40 ± 0.75 (*p* < 0.01) and 10.00 ± 0.73 (*p* < 0.01) days after treatment with 1.0 and 5.0 mg/mL of JPT extract, respectively. Even though the mean lifespan of *C. elegans* was significantly decreased from 9.02 ± 0.47 days to 6.34± 0.47 days after exposure to an oxidative stress inducer (H_2_O_2_). The JPT water extract has failed to extend the lifespan of this nematodes under oxidative stress condition induced by H_2_O_2_.

## Discussion

We have previously shown that some Thai traditional polyherbal formulas including JPT used as tonics or rejuvenators possess remarkable free radical scavenging activities [8]. The consumption of the polyherbal infusion named Tri-Sura-Phon which possessed excellent antioxidant profiles for 8-weeks significantly improves the lipid profile of overweight volunteers [20]. Using a well-established nematode model for lifespan extension, *C. elegans*, we have established scientific evidences for supporting the medicinal use of JPT polyherbal infusion. This work shows that using extraction method mimicking traditional preparation remedies given by traditional practitioners produced the extract with good activity.

The transition metal in particular, ferrous ion plays a key role in the formation of hydroxyl radical via the Fenton reaction, causing oxidative damage to biomolecules such as DNA, lipids, and protein [21]. Our results revealed that water extracts of JPT and its herbal components possess excellent metal chelating ability compared to the ethanol and ethyl acetate extracts. This might be due to the solubility of carbohydrates is higher than that of the pure solvent extracts as have been reported [22]. It has been reviewed that the metal chelating ability of plant-derived polysaccharides are attributed to their uronic acid, sulphate, and carboxyl groups [23]. Even though *P. emblica*, *T. arjuna*, *T. bellirica*, and *T. chebula* showed notable chelating capacities, the water extract of JPT displayed 2–7 times higher metal chelating ability in comparison to its herbal components. JPT showed a fair metal chelating activity compared with a strong chelator, EDTA. Another hypothesized mechanism of chelating ferrous ion by JPT appears to be closely associated with phenolics found in its herbal constituents such as gallic acid, ellagic acid, b-sitosterol, arjungenin, belleric acid, isocorilagin, chebulanin, chebulagic acid, etc. which possess a number of hydroxyl groups [24, 25]. The ferrous ion chelating potential of JPT, therefore, might be caused by its polysaccharide which obtained mainly via water infusion or phenolics derived by both water and organic solvent extractions.

Chain-breaking antioxidant activity is defined as the ability of a compound to inhibit the propagation of oxidizing chain reactions, normally measured through its ability to react with a stable free radical. Based on the antioxidant data obtained via the mixed-mode methods, the water and ethanol extracts of JPT and *P. emblica* displayed notable antioxidant activity. ABTS^•+^ radical is soluble in both water and organic solvents, thereby suitable for testing both hydrophilic and lipophilic compounds. The IC_50_ value of JPT extracts, in particular its ethanol and water extracts were observed to be very low indicating relatively high ABTS^•+^ scavenging efficacy compared to free trolox (positive control). With exception of *T. arjuna*, JPT’s herbal components were also observed to be powerful free radical scavengers against ABTS^•+^. These results are consistent with earlier reports that have proven the free radical scavenging activities of *T. chebula* [24], *T. bellirica*, and *P. emblica* [26], while the activity of *T. arjuna* were mainly reported from its bark [25]. The radical scavenging effects of JPT water and ethanol extracts on DPPH, were found to be 2–3 times less potent than trolox. The activity of *P. emblica* water extract was comparable with the positive control which is consistent with prior knowledge on the antioxidant activities of the fruit of *P. emblica* in in vitro, in vivo [27], and clinical studies [28]. Free radical scavenger potentials of JPT may be caused by its abundant phenolic and flavonoid compounds which are quinic acid, gallic acid, chebulagic acid, ginkgolide C, chebulinic acid, ellagic acid, and rutin. The scavenger activity of the phenolic compounds is attributed to 3′,4′-dihydroxy substitution pattern attached to the flavonoid B-ring or the phenolic hydroxyl group at the C-3 position of the flavonoid C-ring [29].

The excessive accumulation of reactive oxygen species including peroxyl radicals and superoxide anion causes cell death and shortens the lifespans of organisms. Based on the observed result, it was clearly demonstrated that JPT as well as its herbal components are potent peroxyl radical and superoxide anion scavengers in vitro. Similar results were reported from the bark of *T. arjuna* [30], fruits of *T. chebula*, *T. bellirica*, and *P. emblica* [26]. To the best of our knowledge, this is the first report describing in vivo effects of JPT water extract. The mean lifespan of *C. elegans* was considerably improved upon treatment with 1.0 and 5.0 mg/mL of JPT, but the water infusion of JPT may not affect the repair mechanisms of oxidative stress-damaged biomolecules. The effects of this extract on the accumulation of ROS in *C. elegans* as well as the levels of oxidative stress markers such as malondialdehyde, 8-oxo-7,8-dihydro-2′-deoxyguanosine, etc. under the oxidative stress conditions induced by ROS-generating compounds such as paraquat or juglone should be evaluated. Since this water extract revealed promising in vitro free radical scavenging activities, further works on its in vivo free radical scavenging effects and their roles on lifespan and health span extension of *C. elegans* are required. Several studies have confirmed that medicinal plants and plant-derived compounds with free radical scavenging effects remarkably increased the mean lifespan of *C. elegans* under normal and oxidative stress conditions [3–7]. Increasing in the lifespan of *C. elegans*, which had been exposed to paraquat as a superoxide free radical generator was observed after treatment with the extracts of *Ilex paraguariensis* [31]. Only few studies have been done to determine the cytoprotective and life span prolonging effects of JPT’s component. *P. emblica* was found to extend the lifespan and influence some fitness characters in *Drosophila melanogaster* [32]. The bark of *T. arjuna* [33] and fruits of *T. chebula* [34], *T. bellirica*, and *P. emblica* [35] were found to have protective effect against oxidative stress induced cell damage. Despite the well-established in vitro antioxidant capacities of JPT and its herbal compositions, the effect on the life span and their underlining mechanisms remain evasive.

## Conclusion

In conclusion, we have shown that the water extract of JPT, a traditional Thai herbal preparation, extended the life span of *C. elegans* under normal condition which might be related to the peroxyl and superoxide anion scavenging activities of the extract. Additionally, we provided a definitive report on antioxidant activity of JPT and its herbal components including *T. arjuna*, *T. chebula*, *T. bellirica*, and *P. emblica*. According to our data, *P. emblica* could be noted as an active herbal ingredient involved in JPT free radical scavenging properties, while *T. arjuna* and *T. chebula* may act as metal chelating agents in JPT. Taking this information together, JPT water extract exhibited beneficial effects on the survival rate of *C. elegans* under oxidative stress condition and could be further explores as promising plant-based antioxidant.

## Data Availability

All data generated or analysed during this study are included in this published article.

## Abbreviations

ABTS: 2,2′-azino-bis (3-ethylbenzothiazoline-6-sulfonic acid);
DPPH: 2,2-diphenyl-1-picrylhydrazyl
MCA: Metal-chelating activity
MTT: Methyl thiazol tetrazolium
NBT: Nitroblue tetrazolium
ORAC: Oxygen radical antioxidant capacity
TFC: Total flavonoid content
THP: Traditional herbal preparation
TPC: Total phenolic content
TPTZ: Tripyridyl triazine
